# Point of care platelet activity measurement in primary PCI [PINPOINT-PPCI]: a protocol paper

**DOI:** 10.1186/1471-2261-14-44

**Published:** 2014-04-04

**Authors:** Thomas W Johnson, Debbie Marsden, Andrew Mumford, Katie Pike, Stuart Mundell, Mark Butler, Julian W Strange, Ruth Bowles, Chris Rogers, Andreas Baumbach, Barnaby C Reeves

**Affiliations:** 1 Bristol Heart Institute, Bristol Royal Infirmary, Upper Maudlin Street, Bristol BS2 8HW, UK; 2 University of Bristol, Bristol Royal Infirmary, Upper Maudlin Street, Bristol BS2 8HW, UK

**Keywords:** Myocardial infarction, Percutaneous coronary intervention, Antiplatelet therapy, Anti-thrombotic therapy, Platelet function testing

## Abstract

**Background:**

Optimal treatment of acute ST-elevation myocardial infarction (STEMI) involves rapid diagnosis, and transfer to a cardiac centre capable of percutaneous coronary intervention (PCI) for immediate mechanical revascularisation. Successful treatment requires rapid return of perfusion to the myocardium achieved by thromboaspiration, passivation of the culprit lesion with stent scaffolding and systemic inhibition of thrombosis and platelet activation. A delicate balance exists between thrombosis and bleeding and consequently anti-thrombotic and antiplatelet treatment regimens continue to evolve. The desire to achieve reperfusion as soon as possible, in the setting of high platelet reactivity, requires potent and fast-acting anti-thrombotic/anti-platelet therapies. The associated bleeding risk may be minimised by use of short-acting anti-thrombotic intravenous agents. However, effective oral platelet inhibition is required to prevent recurrent thrombosis. The interaction between baseline platelet reactivity, timing of revascularisation and effective inhibition of thrombosis is yet to be formally investigated.

**Methods/Design:**

We present a protocol for a prospective observational study in patients presenting with acute STEMI treated with primary PCI (PPCI) and receiving bolus/infusion bivalirudin and prasugrel therapy. The objective of this study is to describe variation in platelet reactivity, as measured by the multiplate platelet function analyser, at presentation, the end of the PPCI procedure and 1, 2, & 24 hours post-procedure. We intend to assess the prevalence of high residual platelet reactivity within 24 hours of PPCI in acute STEMI patients receiving prasugrel and bivalirudin. Additionally, we will investigate the association between high platelet reactivity before and after PPCI and the door-to-procedure completion time.

This is a single centre study with a target sample size of 108 participants.

**Discussion:**

The baseline platelet reactivity on presentation with a STEMI may impact on the effect of acute anti-thrombotic and anti-platelet therapy and expose patients to a heightened risk of bleeding or ongoing thrombosis. This study will define the baseline variation in platelet reactivity in a population of patients experiencing acute STEMI and assess the pharmacodynamic response to combined treatment with bivalirudin and prasugrel. The data obtained from this trial will be hypothesis generating for future trials testing alternative pharmacotherapies in the acute phase of treatment for STEMI.

**Trial registration:**

This study has approval from Wiltshire research ethics committee (10/H0106/87) and is registered with current controlled trials (http://www.controlled-trials.com/ISRCTN82257414).

## Background

Primary percutaneous coronary intervention (PPCI) is accepted as the optimal treatment for acute ST-elevation myocardial infarction (STEMI) [1]. Restoration of myocardial perfusion is achieved by thrombo-aspiration, passivation of the culprit lesion with stent scaffolding and systemic inhibition of thrombosis and platelet activation.

Platelet activation occurs early on in a cascade of events leading to coronary arterial occlusion and STEMI. Therefore, anti-platelet therapy is fundamental to the successful re-canalisation and subsequent reperfusion of the myocardium. The nature of STEMI treatment is time critical and currently anti-thrombotic treatment involves using both oral and intravenous agents to confer immediate and long-term anti-thrombotic effects. Significant advances in the inhibition of platelet activity have occurred in recent years and current data supports the use of increasingly potent anti-thrombotic agents and platelet inhibitors to limit the risk of future cardiac events, including mortality. However, increasingly potent and advanced pharmacotherapy comes at a financial cost and with an increased risk of complications, particularly bleeding. A balance has to be achieved between effective suppression of thrombosis, prevention of significant bleeding and cost.

The Bristol Heart Institute (BHI) anti-thrombotic protocol for the treatment of STEMI was revised in 2010. The revision implemented conversion from using un-fractionated heparin (UFH) with glycoprotein 2b/3a receptor inhibitors (GPI) to using bivalirudin monotherapy (a drug with rapid onset/offset and excellent bleeding profile) in the catheter laboratory and, additionally, conversion from clopidogrel to prasugrel (a drug with a consistent and potent platelet inhibitory effect) thereafter. Prasugrel is administered to patients on arrival at the BHI prior to undertaking angiography and PPCI. Use of bivalirudin has been shown to offer equivalent benefit in terms of limiting major adverse cardiac events (MACE), with a reduction in cardiac mortality and bleeding versus UFH plus GPI [2]. Prasugrel is a potent inhibitor of the platelet ADP receptor P2Y12. It was tested against clopidogrel in the TRITON-TIMI 38 trial and demonstrated a significant reduction in a composite outcome of cardiac mortality, non-fatal myocardial infarction and non-fatal stroke in the treatment of STEMI [3].

The use of prasugrel and bivalirudin in the acute treatment of STEMI offers excellent anti-platelet and anti-thrombotic effect [2,3]. The combination of prasugrel with bivalirudin should confer increased protection against early thrombotic events, as prasugrel has been demonstrated to offer faster inhibition of platelet reactivity compared to clopidogrel [4]. However, it should be noted that the HORIZONS-AMI trial revealed an increased rate of very early stent thrombosis at 24 hours with bivalirudin monotherapy. Reassuringly, at 30 days the stent thrombosis rate was not statistically different in both treatment groups [5]. This transient early negative outcome with bivalirudin most likely relates to the pharmacokinetics of the drug, and the protocol of administration. Bivalirudin is administered intravenously, with an initial bolus (0.75 mg/kg) and then an infusion (1.75 mg/kg/h) until the completion of the angiography/PCI procedure. Bivalirudin has a half-life of ≈ 25 minutes and, therefore, thrombin activity is restored fairly rapidly when the infusion stops. The increase in acute stent thrombosis may signal a gap in anti-thrombotic protection arising from a gap between the waning anti-thrombin effect of Bivalirudin and the onset of platelet inhibition from clopidogrel. Prasugrel has been demonstrated to achieve effective platelet inhibition (>40% inhibition of platelet activity) within 30 minutes of oral ingestion in patients with stable angina, compared with 2 hours for equivalent inhibition with clopidogrel [4]. Despite impressive pharmacodynamic data in a stable population, recent studies of patients presenting with STEMI, treated with prasugrel, demonstrate a delayed inhibition of platelet function, extending beyond 2 hours [6,7].

The 2010 revision of the BHI anti-thrombotic protocol (preceding data relating to delayed prasugrel effect in STEMI) assumes that combined prasugrel and bivalirudin therapy for the treatment of STEMI will provide effective anti-platelet and anti-thrombotic inhibition, minimising the risk of acute stent thrombosis and bleeding. The validation of this assumption in ‘real-world’ STEMI patients has not yet been tested.

### Aim

We hypothesise that continued reductions in the “door to balloon” time (time from hospital admission to achieving an open artery in the catheter laboratory) may result in some patients undergoing PPCI with incomplete P2Y12 blockade, leading to an increase in the risk of early thrombotic events. We speculate that the rapid restoration of coronary flow achieved with PPCI may offer inadequate time to achieve platelet inhibition with prasugrel, prior to the loss of the anti-thrombin effect of bivalirudin.

Therefore, the objectives of this study are to describe the prevalence of high residual platelet reactivity within 24 hours of PPCI in acute STEMI patients receiving a prasugrel/bivalirudin anti-platelet regime and, to assess the association between high residual platelet reactivity after PPCI and door-to-procedure completion time and baseline platelet reactivity before PPCI.

## Methods

### Study design and duration

The PINPOINT-PPCI (A study of platelet inhibition, using a ‘point of care’ platelet function test, following primary percutaneous coronary intervention for ST elevation myocardial infarction) study is a single centre, prospective, observational study. For each patient, the study follow-up period is 30 days. The study is anticipated to last 24 months, including a 6 month run-in period for catheter laboratory staff training on the multiplate analyser. Enrolment to the trial commenced in June 2011.

### Selection criteria

Patients presenting to the BHI, a regional heart attack centre, are considered for inclusion in the study. Eligibility for the trial requires presentation with an acute STEMI with planned treatment by PPCI. Participants are excluded if there is evidence of active bleeding or bleeding diathesis, previous history of cerebrovascular event, weight <60 kg, use of clopidogrel, prasugrel or ticagrelor within 7 days of presentation, or haemodynamic instability.

### Recruitment

Patients are identified on arrival at the BHI when presenting with STEMI. The operator (interventional cardiologist) explains the PINPOINT-PPCI study to eligible patients and obtains verbal assent at the time of procedural consent. Formal written study consent is obtained within 24 hours of recruitment, following a period of recovery from the PPCI procedure and after a minimum of 4 hours to review/discuss the details of the study, as described in a patient information sheet (PIS).

Patients receive a 60 mg loading dose of prasugrel as soon as possible on arrival to the hospital (emergency room or cathlab). A loading dose of 300 mg of Aspirin is either administered in the community or on arrival to hospital. Bivalirudin is commenced at the start of the PCI (0.75 mg/kg bolus followed by 1.75 mg/kg/h infusion during the PCI). Use of unfractionated heparin is discouraged and patients are excluded if requiring use of a GPI or continuation of the bivalirudin infusion post-procedure.

### Study measurements

The primary outcome measure for the study is the ADP receptor platelet function assessment measured in peripheral ‘whole’ blood using a multiple electrode analyser (MEA – Multiplate platelet function analysis). Secondary outcome measures of platelet function include assessment of the arachidonic acid pathway, thromboxane receptor and thrombin related platelet activation.

**Figure 1:**
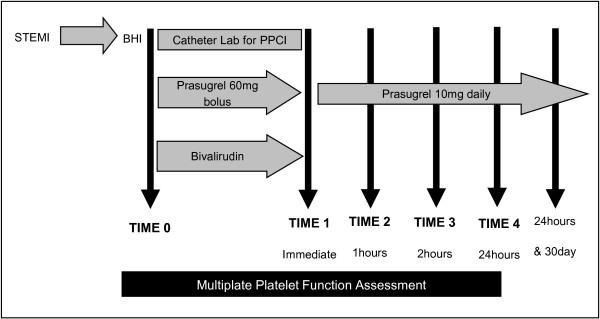
Study schema.

Platelet function (measured in peripheral ‘whole’ blood, see above) will be assessed on arrival at hospital, immediately after completion of the PPCI and 1, 2 and 24 hours after the completion of the procedure (Figure 1). Multiple measurements will facilitate the generation of a profile of platelet function from presentation through the post-procedural period, describing the combined waning effect of bivalirudin and increasing platelet inhibition by prasugrel. These same platelet function measurements will enable assessment of the impact of initial platelet reactivity and the door-to-procedure completion time on effective platelet inhibition, as measured by ADP receptor platelet function.

Additional secondary outcome measures include the incidence of adverse clinical events at 24 hours and 30 days, including major adverse cardiac events (MACE – a composite of target vessel revascularisation, target lesion revascularisation, non-fatal myocardial infarction, and cardiac death), bleeding complications (using the trials in myocardial infarction (TIMI) major and minor bleeding criteria [8] and bleeding academic research consortium (BARC) definition for bleeding [9]), and stent thrombosis (Academic research consortium (ARC) definition [10]).

### Confounding variables

We are measuring and recording variables that may potentially confound the associations of interest. These variables are: timing of symptoms/presentation relative to initiation of treatment, age, co-existent diabetes, and prior treatment with aspirin. All of these variables affect the characteristics of the index MI and may also influence door-to-balloon time and platelet reactivity on presentation.

### Procedure/Laboratory methods

Platelet function is assessed at baseline and multiple time points after completion of the PPCI procedure, i.e. 0, 1, 2 and 24 hours. Multiplate platelet function analysis has been well validated for assessment of the anti-platelet effect of ADP receptor inhibitors and aspirin, offering very useful information regarding the future risk of significant adverse events [11].

MEA-derived platelet function is assessed using ‘whole blood’ samples. However, platelets are very sensitive to mechanical disruption and have a tendency to aggregate with time after obtaining the blood sample, impairing assessment. Consequently, platelet function testing requires careful sampling and timely analysis. MEA is a ‘point of care’ system and, therefore, we have installed a multiplate platelet function analyser in the catheter laboratory, enabling us to undertake immediate analysis of blood samples.

The MEA test requires a single 3 ml blood sample. At baseline, the sample is withdrawn from the arterial sheath (1 x 3 ml Hirudin sample) at the time of routine blood sampling, before starting the PPCI. The second sample (Time 1) is also taken from the arterial sheath at the end of the procedure. Subsequent samples (Times 2–4) are taken 1, 2 & 24 hours following completion of the PPCI using a standard venepuncture technique. Where ever possible venepuncture is scheduled to co-incide with clinically required phlebotomy. Platelet activity (arachidonic acid, thrombin receptor, thromboxane receptor & ADP-receptor activation assessment – ASPI, TRAP, U46119 & ADP tests) in the samples is assessed immediately using the multiplate analyser.

### Adverse event monitoring

Serious and other adverse events are recorded and reported in accordance with the International Conference for Harmonisation of Good Clinical Practice (ICH GCP) guidelines and the Sponsor’s Research Related Adverse Event Reporting Policy. However, the only research procedures that patients are required to undergo for this study are venepuncture (for blood samples).

### Sample size calculation

The primary analysis will be a mixed model regression (see below) to test whether or not door-to-procedure completion time and platelet function on arrival at hospital are significant predictors of platelet function after PPCI (key predictors of interest). In a multiple regression model, the analogous quantity to a “target difference” (for a binary predictor) is the target increase in the proportion of the variance explained. In PINPOINT, this target [that the study was powered to detect] was a 5% increase in the proportion of the variance explained by the model with and without the predictors of interest (i.e. 25% for the full model, and 20% for the model excluding the key predictors of interest).

Two key predictors will be tested. Therefore, a significance level of 0.025 will be adopted (Bonferroni correction) giving a nominal hypothesis test at p < 0.05 for each predictor. For 80% power, the above assumptions require a target sample size of 135. This target sample size needs to be further adjusted by the relative efficiency of the analysis, i.e. to take into account the three repeated measures. Estimating the relative efficiency requires further assumptions about the correlations between the repeated measures; for correlations ranging from 0.5 to 0.8, the relative efficiency varies from 1.25 to 1.50. Taking the lower estimate of relative efficiency gives a final target sample size of 108 (i.e. 135/1.25).

### Statistical analysis

The primary analysis will be a mixed model regression to take into account the four repeated measures of platelet function at the end of the procedure, 1, 2 and 24 hours after completion of PPCI. The analyses will adjust for the potential confounding factors described above. The two key predictors will be tested in the final model, namely the effects of (a) door-to-procedure completion time and (b) platelet function on arrival at the hospital.

Secondary outcomes will be analysed using logistic regression, adjusting separately for each of the key predictors. Other potential confounding factors will only be adjusted for in models if there have been sufficient numbers of outcome events that estimates can be estimated reliably, i.e. over-adjustment is not considered to be a potential problem.

The numbers of adverse events (both serious and non-serious) will be described.

### Ethics

Ethics review of the protocol for the study and other study-related essential documents (e.g. PIS, consent form) was undertaken by a UK NHS Research Ethics Committee (REC). This study does not raise any substantive ethical issues since participants are receiving standard NHS care and are having a research procedure that carries very little risk.

This study is being conducted in accordance with the European Union Directive 2001/20/EC on clinical trials, the Medicine for Human Use (Clinical Trial) Regulations 2004, the International Conference for Harmonisation of Good Clinical Practice (ICH GCP) guidelines and the Research Governance Framework for Health and Social Care.

### Study limitations

The initial study design had anticipated a ‘run-in’ period of catheter laboratory staff training and subsequent recruitment of patients at any time during the day or night during the working week. However, the Multiplate platelet function analyser is not a true ‘point of care’ test, since it requires operators to be trained and takes approximately 10 minutes to process a sample. The multiple measurements required for each patient in the study has precluded involvement of the staff in the catheter laboratory and, instead, we are relying on dedicated laboratory staff. The unpredictable nature of patients presenting with STEMI combined with a need for trained personnel to undertake the test has limited recruitment to periods when laboratory staff are available; this has mainly restricted enrolment to Monday-Friday, 0800–1600 hours. Furthermore, the time constraints associated with multiple measures of platelet function resulted in rationalisation of the study protocol, which initially included a 4 hour post-procedure timepoint. Although, additional timepoints would provide more information regarding the kinetic of platelet inhibition, it was felt that the logistical implications would jeopardise recruitment to the study. Patients with thrombotic complications during PPCI, requiring ongoing intravenous anti-thrombotic/anti-platelet therapy (physician discretion), are excluded from the trial due to the impact that ongoing intravenous therapy has on the platelet function assessment. This group of patients merit further investigation.

The emergency nature of presentation with STEMI prevents a standard consenting process for participation in clinical trials. Verbal assent with written consent delayed until after acute treatment is an accepted method of recruitment in the emergency setting and has been successfully adopted in our department for a number of trials. Both staff and patients accept the process and to date, all patients assenting to the study have subsequently completed written consent.

## Discussion

This study is assessing the acute variability of platelet reactivity at the time of presentation with STEMI and the impact of accelerated ‘door-to-balloon’ time on the platelet inhibitory effect of anti-thrombotic and anti-platelet agents within the first 24 hours of care. Debate continues regarding the acute safety of bivalirudin monotherapy in the treatment of STEMI [12-14]. Despite evidence of rapid platelet inhibition with prasugrel in stable patients, and encouraging early reports of their combined use [15], it is not clear if the combination of prasugrel and bivalirudin is sufficient to prevent acute stent thrombosis. It is possible that the baseline platelet reactivity, on presentation with a STEMI, impacts on the effect of acute anti-thrombotic and anti-platelet therapy and exposes patients to a risk of bleeding or ongoing thrombosis. We anticipate that demonstration of the baseline variation in platelet reactivity in patients experiencing acute STEMI and definition of the pharmacodynamic response to combined treatment with bivalirudin and prasugrel will facilitate optimisation of pharmaco-therapeutic strategies utilised in the acute phase of treatment for STEMI.

### Trial status

Recruitment to the PINPOINT study is nearing completion, with 100patients recruited. It is anticipated that the study results will be available for dissemination later in 2013.

## Abbreviations

STEMI: ST elevation myocardial infarction; PCI: Percutaneous coronary intervention; PPCI: Primary percutaneous coronary intervention; BHI: Bristol Heart Institute; UFH: Unfractionated heparin; GPI: Glycoprotein 2b/3a inhibitor; MACE: Major adverse cardiovascular events; ADP: Adenosine di-phosphate; MEA: Multiple electrode analyser; TIMI: Trials in myocardial infarction; ARC: Academic research consortium.

## Competing interests

The authors declare that they have no competing interests.

## Authors’ contributions

TJ conceived the study, AM, SM, MB, BR, AB & TJ participated in the design of the study, and writing of the protocol. TJ, DM, AM, KP, RB, JWS, BR, CR & AB contributed to the writing of the manuscript and all authors have read and approved the final manuscript.

## Pre-publication history

The pre-publication history for this paper can be accessed here:

http://www.biomedcentral.com/1471-2261/14/44/prepub
